# The Balanced Scorecard: a tool to monitor IPL curriculum implementation: January 2020

**DOI:** 10.15694/mep.2020.000010.1

**Published:** 2020-01-14

**Authors:** Maree O'Keefe, Adam Montagu, Frank Donnelly, Tamara Page, Helena Ward

**Affiliations:** 1 University of Adelaide

**Keywords:** Curriculum, Interprofessional, IPL, Delphi survey, Quality assurance

## Abstract

This article was migrated. The article was marked as recommended.

**Introduction**: Implementing interprofessional learning (IPL) in health profession curriculum is difficult despite widespread acknowledgement of the importance of interprofessional collaborative health care practice. The aim of this study was to develop a balanced scorecard using a Delphi technique to document and monitor implementation of IPL in a faculty of health and medical sciences.

**Methods/Results**: Twenty-four academic teachers and health service clinical supervisors completed two electronic questionnaires as part of a two stage Delphi survey. Consensus (70% agreement/disagreement) was achieved for 27/36 items in round one and for all 10 items in round two. Ten performance metrics were subsequently identified.

**Discussion**: The Delphi was an efficient and effective method for identifying performance metrics for monitoring faculty IPL implementation. With a strong focus on learning outcomes and assessment, the scorecard will enable the faculty to formally monitor implementation of our IPL strategy over time. A follow up process of identifying data sources for reporting against each of the scorecard items has already highlighted gaps in our current practices, predominantly in staff professional development and assessment.

## Introduction

As interprofessional learning (IPL) brings together multiple health professions, there is inherent complexity in curriculum design and delivery. Not only do specific IPL competencies need to be achieved, but individual professional competencies are also required, sufficient to satisfy external accreditation agencies. Articulation of IPL mission, vision and value statements is a critical first step in curriculum design. However, unless these are translated into activities that are then monitored and sustained, mission and vision statements can remain aspirational, especially in contexts as complex as IPL ( Farnsworth *et al.*, 2015). An educational balanced scorecard ( Huntington, Dick and Ryder, 2018) is a performance management tool that facilitates translation of educational mission into action, gives objective evidence of progress, and provides a firm basis for strategic decision making. In particular, such a tool can monitor the success of initiatives to integrate IPL into the core health profession curriculum. To realise our vision for IPL, the aim of this study was to develop a balanced scorecard using a Delphi technique.

## Methods

The study was undertaken at the University of Adelaide, South Australia, Faculty of Health and Medical Sciences between June and December 2019. The faculty offers undergraduate degrees in dentistry, health and medical sciences, medicine, nursing, oral health and psychology. A suite of allied health degrees will also be offered from 2021. The study was approved by the University of Adelaide Human Research Ethics Committee (approval number H-2019-081).

The Delphi technique is a method of systematically gathering information and building consensus among key experts and/or stakeholders ( de Villiers, de Villiers and Kent, 2005). Following an initial survey of opinions on a series of statements, outcomes of this first round are provided to all participants who are then re-surveyed.

An IPL taskforce of key experts was established to initiate the study. The taskforce comprised eight members of academic faculty staff (including the authors) each of whom had responsibility for IPL curriculum development and delivery in one or more of the faculty undergraduate health degrees. The principle role of the taskforce was to identify potential items for inclusion in round one of the Delphi survey, to review the results, to agree items for inclusion in round two and to finalise a set of metrics for the scorecard. A broader stakeholder group was identified also. This comprised academic teachers and health service clinical supervisors with involvement in the faculty teaching programs as course coordinators or similar leadership roles.

Stakeholders and taskforce members were invited to complete two electronic questionnaires. The second questionnaire was available one month after the first and each were open for two weeks. Participants were advised that completion of the online questionnaire would constitute consent to participate. Round one items were derived from the faculty IPL vision and core values and addressed four key strategic domains: curriculum, student outcomes, staff engagement, and community and staff value. Round one items were rated on a 5 point Likert scale (strongly agree/ agree/ neutral/ disagree/ strongly disagree). Respondents could also provide comment on each item. A consensus outcome was confirmed when 70% or more of respondents strongly agreed/agreed or disagreed/strongly disagreed with that particular item. Items without consensus after round one were modified in light of respondent comments and included in round two where a simple agree/ disagree response was sought. A report of the outcomes of round one was provided to all recipients of the round one invitation together with the round two questionnaire. Participants were asked to indicate their discipline background, but no other demographic information was sought. Participants could also provide free text comments for any item.

## Results/Analysis

Twenty-four academic teachers and health service clinical supervisors completed each round (response rate 22%). Participant disciplines included dentistry/oral health, medicine, nursing, public health, psychology, and medical sciences. Round one comprised 36 items with consensus achieved for 27 items. Round two comprised 10 items with a consensus outcome achieved for all. The final outcome was a set of 10 performance metrics across the four domains ( Table 1). Further information regarding the questions is available at https://adelaide.figshare.com/articles/Final_Delphi_outcomes_pdf/11356880.

**Table 1.** Faculty IPL scorecard

Domain	Metric
Curriculum	Number of clinical * degrees with an IPL learning outcome in at least one component course at each year level
	Number of faculty courses with IPL learning outcomes
	% allocation of assessment marks for IPL learning outcomes assessment in clinical courses
Student outcomes	Number of OSCE examinations with IPL content
	Number of students achieving the 8 IPL competencies ** by graduation in accredited clinical programs
Staff engagement	Number of academic staff who have received IPL induction
	Number of academic staff who have participated in IPL training
	Reference to IPL is made in the Faculty Research Strategy
	Number of program coordinator reports on IPL activities in programs board minutes
Community and staff value	Number of assessment tasks where patients and standardised patients contribute to the assessment of students

* Dentistry, medicine, nursing, oral health

** O’Keefe, M., Henderson, A. and Chick, R. (2017) ‘Defining a set of common interprofessional learning competencies for health profession students,’ *Medical Teacher*, 39(5), pp. 463-468. https://doi.org/10.1080/0142159X.2017.1300246

## Discussion

The Delphi proved to be an efficient and effective method for identifying performance metrics for monitoring faculty IPL implementation with a strong focus on learning outcomes and assessment emerging. The initial phase of identifying items for inclusion prompted careful consideration of the current faculty vision and strategic priorities for IPL. Congruency was evident between the existing vision and high level strategic priorities. However, the process of identifying specific performance metrics for the present study gave clarity to the individual areas that required further attention so the vision could be realised. This was brought into particularly sharp focus when we turned our minds to the nature of the data sources required to address each metric. Identifying specific data sources for reporting against each of the scorecard items highlighted gaps in our current practices, especially with assessment of specific IPL learning outcomes and staff professional development.

To the best of our knowledge this is a novel approach to achieving and monitoring implementation of IPL curriculum. A highly inclusive approach was used to identify potential participants. This was in part a deliberate profile raising activity for IPL and viewed as an opportunity to characterise IPL as a component of core contemporary health profession curriculum. Although response rates were low as a percentage of invitees, the number of participants was appropriate for a Delphi survey and all invitees were equally qualified and experienced to participate. Along with the outcomes of round one, the final set of metrics were circulated to all original invitees for information and comment. We are aware that several important stakeholder groups were not included in this first iteration of the scorecard. It is intended that subsequent evaluation will include the perspectives of students and members of the lay community.

## Conclusion

The Delphi was an efficient and effective way of identifying performance metrics for monitoring faculty IPL implementation. The balanced scorecard was easily embedded into the faculty educational governance systems with the initial application being a baseline assessment of faculty performance against each item. The process of identifying data sources for reporting against each of the scorecard items has already highlighted gaps in our current practices, predominantly in staff professional development and assessment. The report will now be run on an annual basis to document and chart progress with further feedback and refinement if required. A future iteration could also see the development and inclusion of specific targets and goals.

## Take Home Messages

• The Delphi provided an efficient and effective way of identifying performance metrics for monitoring faculty IPL implementation.
• The scorecard has a strong focus on learning outcomes and assessment of IPL
• Scorecard development triggered review of current faculty IPL vision and strategic priorities
• The balanced scorecard was easily embedded into the faculty educational governance systems
• Identifying data sources for reporting against each of the scorecard items highlighted gaps in current practice

## Notes On Contributors

Maree O’Keefe: Director Curriculum, Faculty of Health and Medical Sciences, University of Adelaide. ORCID ID: https://orcid.org/0000-0002-0371-5322

Adam Montagu: Director Adelaide Health Simulation, Faculty of Health and Medical Sciences, University of Adelaide.

Frank Donnelly: Acting Head, Adelaide Nursing School, Faculty of Health and Medical Sciences, University of Adelaide. ORCID ID: https://orcid.org/0000-0001-7675-9505

Tamara Page: Pre-registration Program Coordinator, Adelaide Nursing School, Faculty of Health and Medical Sciences, University of Adelaide. ORCID ID: https://orcid.org/0000-0002-0771-7034

Helena Ward: Senior Lecturer in Assessment, Adelaide Medical School, Faculty of Health and Medical Sciences, University of Adelaide. ORCID ID: https://orcid.org/0000-0002-3831-1205
