# Supplementary material for: The Balanced Scorecard: a tool to monitor IPL curriculum implementation: January 2020
Source: MedEdPublish (2016). 2020 Jan 14;9:10. [Version 1] doi: 10.15694/mep.2020.000010.1 (PMC10712626; doi:10.15694/mep.2020.000010.1)
Supplement: Peer review history

# Reviewer report: Reviewer response for version 1

**DOI:** 10.21956/mep.19978.r30383

**Published:** 2020-05-11

**Authors:** Ken Masters

**Affiliations:** 1 Sultan Qaboos University

This review has been migrated. The reviewer awarded 3 stars out of 5 An interesting read on the important topic of developing an IPL curriculum.I have just a few points:• Given the very low response rate (itself, a concern that really should be noted as a limitation in the Discussion), the statement that “Participant disciplines included dentistry/oral health, medicine, nursing, public health, psychology, and medical sciences” is too broad as the only description of the response participants. One needs to see a table of the number of representatives from each group (vs. the total number of people in each group) to determine the overall representativity of the sample.• Similarly, the statement “Round one comprised 36 items with consensus achieved for 27 items. Round two comprised 10 items with a consensus outcome achieved for all” is far too brief. A reader does need to seeo a list of the 36 itemso an indication of the 27 that received consensus, ando a description of the process that took these 27 down to 10. I see that the authors do supply a link to a pdf file that is on the institutional website, but that information is not an optional extra to the paper; it (and fleshed out in some more detail) is part of the paper’s data, and really is crucial to the paper’s value. As a result, it really should be included in the paper, either in the main body of the text, or as Appendices or as Supplementary Files. That way, the paper can more accurately be assessed, and that information will also be stored in the Journal’s permanent archiving and indexing systems, and not subject to administrative decisions by third parties.So, I look forward to a Version 2 of the paper in which this extra information is given, so that a more accurate assessment of the research can be made.

Reviewer Expertise:

NA

No decision status is available

# Reviewer report: Reviewer response for version 1

**DOI:** 10.21956/mep.19978.r30384

**Published:** 2020-03-11

**Authors:** Trevor Gibbs

**Affiliations:** 1 AMEE

This review has been migrated. The reviewer awarded 4 stars out of 5 It is interesting that after nearly thirty years of suggesting that IPL is an important / essential element within health professions education, we are still discussing its implementation. One of the major issues is exactly what the authors of this paper state- mission and vision statements are often aspirational rather than active and practical. I enjoyed reading this paper and believe it describes a novel approach to clarification of the organisational management of IPL in the curriculum. It is a novel but simple approach which has resulted in clarity of purpose. It is sad to see such a lowish faculty response to such an important issue, but I am not convinced that this affected the overall result. I suppose the future of this piece of research is based upon seeing if this approach not only clarifies the position of IPL but results in a strong IPL element within the curriculum and achievement of its educational outcomes.I would recommend this paper to all tasked with implementing IPL into their curriculum.

Reviewer Expertise:

NA

No decision status is available
